# Effect of Magnetic Fields on the Development of the Larvae of the Jaguar Cichlid (*Parachromis managuensis*, Günther, 1867) and the Green Terror (*Andinoacara rivulatus*, Günther, 1860)

**DOI:** 10.3390/ani15131824

**Published:** 2025-06-20

**Authors:** Radosław Piesiewicz, Agata Korzelecka-Orkisz, Krzysztof Formicki

**Affiliations:** Department of Hydrobiology, Ichthyology and Biotechnology of Animal Reproduction, West Pomeranian University of Technology, 70-310 Szczecin, Poland; agata.korzelecka-orkisz@zut.edu.pl (A.K.-O.); krzysztof.formicki@zut.edu.pl (K.F.)

**Keywords:** fish, fish larvae, magnetic field, survival rate

## Abstract

This article has examined the effects of magnetic fields with intensities of 1 mT, 3 mT, and 5 mT on two species of cichlid fish (family Cichlidae). This study focused specifically on assessing the influence of these magnetic fields on larval development, the survival rate, and the occurrence of deformities in the larvae of the jaguar cichlid (*Parachromis managuensis*) and the green terror (*Andinoacara rivulatus*). The analysis of the study results indicates that the effects of magnetic field exposure depend both on its intensity and on the fish species being studied. Magnetic fields, depending on the applied intensity, can influence changes in body size as well as the size of the yolk sac. In jaguar cichlid larvae, increased survival was observed in the groups exposed to the magnetic field; however, this was accompanied by a higher number of deformities compared with the control group. In the case of the green terror, the highest survival rates were recorded in the control group and in the group exposed to a magnetic field of 3 mT, while increased numbers of deformities were also noted in the exposed groups. The obtained results may be of significant importance for ornamental fish aquaculture, where appropriately selected magnetic field parameters could support larval growth and survival, reducing losses in breeding.

## Simple Summary

Research on the effects of magnetic fields on fish has been conducted for several decades and constitutes a significant area of interest for scientists. The results of those studies to date indicate that magnetic fields play a crucial role in the lives of organisms on Earth. The aim of this study is to investigate the influence of magnetic fields on the larvae of two cichlid species (*Parachromis managuensis* and *Andinoacara rivulatus*). This experiment focused on evaluating the effects of the magnetic field on body size, eye size, and the rate of yolk sac resorption and determining the impact of the magnetic exposure on larval survival and the incidence of deformities. The findings from this study may hold significant implications for ornamental fish aquaculture, where appropriately calibrated magnetic field exposure could potentially enhance larval growth and survival, thereby reducing production losses.

## 1. Introduction

Studies on the effects of magnetic fields on fish have been conducted for several decades; however, the majority of these investigations have focused on the geomagnetic field’s role in spatial orientation, particularly in migratory species [1,2,3]. In the present study, attention was directed toward the effects of artificially generated magnetic fields with intensities of 1 mT, 3 mT, and 5 mT on the development of ornamental fish larvae. These field strengths are approximately 20 to 100 times higher than the Earth’s natural magnetic field, which is around 50 µT (0.05 mT). Due to substantial differences in field intensity, the effects of applied magnetic stimuli should be considered a distinct biological phenomenon unrelated to the spatial orientation mechanisms triggered by the geomagnetic field. To date, there is a lack of detailed studies investigating the effects of strong magnetic fields on the early developmental stages of fish—including eggs and larvae—particularly in the context of their use in large-scale aquaculture systems [4]. Current knowledge in this area remains limited and calls for further in-depth research.

Two cichlid species were selected for this study, the jaguar cichlid (*Parachromis managuensis*) and the green terror (*Andinoacara rivulatus*), as despite their close taxonomic relationship, they differ in many respects. The jaguar cichlid is widely distributed in Central America, from the Ulua River in Honduras to the Matina River in Costa Rica [5,6]. It inhabits lakes, rivers, and estuaries characterized by low water flow and average water temperatures ranging from 25 °C to 30 °C [7,8,9,10,11,12]. In its natural environment, this species can grow to up to 65 cm [13]. The jaguar cichlid exhibits high resistance to unfavorable environmental conditions, including elevated water temperatures, reduced oxygen levels, and fluctuations in salinity [7,14,15].

The green terror is found only in South America, inhabiting waters in Ecuador and Peru [16,17,18,19]. This endemic species lives in shallow, warm, and slow-flowing rivers, selecting coastal zones with large stones for spawning, which serve both as a substrate for egg deposition and as shelter [20]. In the wild, this species grows to up to 20 cm. Due to its sensitivity to water pollution, a decline in wild populations has been observed, and without intervention, this could lead to extinction [21,22].

Previous studies have shown that magnetic fields can influence embryogenesis. Analysis of directional responses in fish embryos of various species has demonstrated that their symmetry axis aligns at a specific angle in relation to both the Earth’s geomagnetic field and artificially generated magnetic fields [23,24,25,26,27,28]. It has also been shown that magnetic fields affect the course of embryogenesis in cichlid species—*A. rivulatus* and *P. managuensis*—by accelerating the process [29]. Moreover, magnetic fields have been found to significantly affect embryonic motor activity: for instance, the heartbeat of embryos exposed to a magnetic stimulus increases until a stable rhythm is reached [30]. Magnetic fields have also been shown to influence the distribution of melanin in melanophores in embryos’ skin [31].

The effects of magnetic fields on fish larvae have also been studied. Formicki and Winnicki [24] showed that magnetic fields acting on the incubating larvae of sea trout (*Salmo trutta*) and rainbow trout (*Oncorhynchus mykiss*) affected their sizes. A prolonged incubation time resulted in hatched larvae that were longer and heavier than those in the control group. Similarly, Krylov [32] demonstrated that magnetic fields influence changes in the body lengths and masses of roach (*Rutilus rutilus*) larvae during incubation. Fey [33] reported that magnetic fields affect the rate of yolk sac resorption in larvae—higher magnetic-flux-density accelerated resorption.

Numerous scientific reports indicate that fish larvae of various species may respond to fluctuations in the Earth’s magnetic field. Coral reef fish are capable of using changes in magnetic flux density for spatial orientation and for selecting settlement sites [34,35,36,37]. Studies of haddock (*Melanogrammus aeglefinus*) and Atlantic cod (*Gadus morhua*) larvae have shown that magnetic fields negatively affect their swimming speed and mean acceleration, although no impact was observed on the spatial distribution of the larvae [38,39]. Research by Putman [40] confirmed that magnetic fields can also influence the hatching larvae of Chinook salmon (*Oncorhynchus tshawytscha*), which were observed to orient themselves toward a generated magnetic field when leaving the nest.

Despite growing scientific interest in the role of magnetic fields during various stages of ontogeny, knowledge about their effects on morphological changes in fish larvae remains very limited. Existing studies primarily focus on commercially farmed species, while no research has been conducted on the impact of magnetic fields on the development of ornamental cichlid larvae. The species *P. managuensis* and *A. rivulatus* were selected due to their increasing popularity in ornamental aquaculture and their distinct ecological and behavioral traits, which may influence their sensitivity to magnetic fields. Both species exhibit high larval survival in captive conditions and are frequently bred under artificial settings, making them suitable models for evaluating the potential impact of magnetic factors in intensive rearing systems.

In recent years, the ornamental fish aquaculture sector has experienced dynamic growth. In 2023, the global ornamental fish market was valued at 5.95 billion USD, with projections suggesting an increase to 11.69 billion USD by 2032 [41]. To effectively breed these fish and protect wild populations, it is essential to develop and support their aquaculture through research that expands our understanding of their biology and can be applied in practice to improve breeding outcomes.

The aim of this study is to investigate the influence of a magnetic field on larvae of two cichlid species (*P. managuensis* and *A. rivulatus*). The experiment focused on evaluating the effects of the magnetic field on body size and eye size, the rate of yolk sac resorption, and determining the impact of magnetic exposure on larval survival and incidence of deformities. These findings are intended to assess the potential positive or negative effects of magnetic fields on cichlid larvae and support the application of magnetic fields in ornamental fish aquaculture to minimize losses among both larvae and juveniles.

## 2. Materials and Methods

This study was conducted in an isothermal laboratory with a stable, controlled temperature. The experiments were carried out first on the eggs and subsequently on the larvae of two cichlid species (Cichlidae): the jaguar cichlid (*P. managuensis*) and the green terror (*A. rivulatus*).

**Figure 1:**
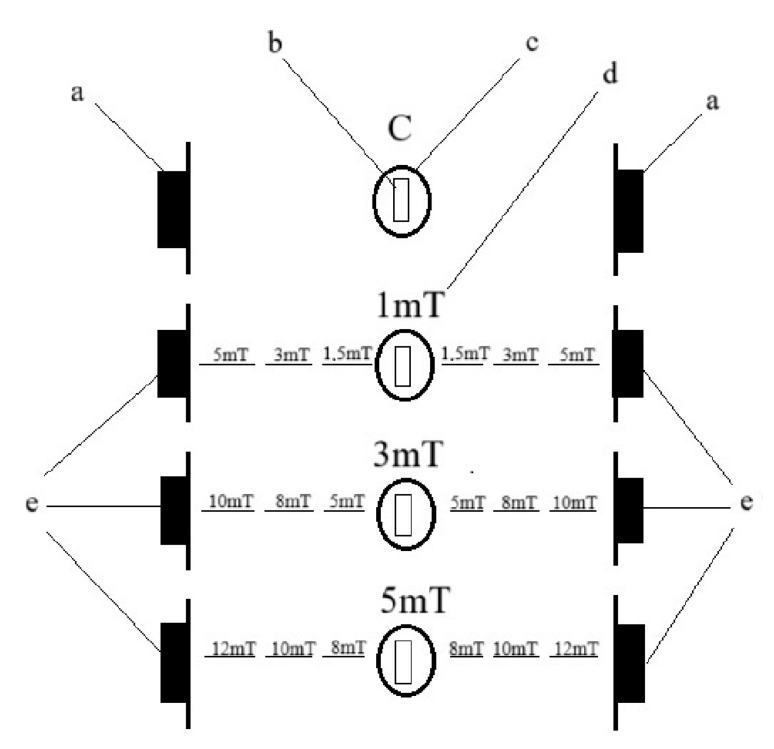
Diagram of the experiment showing the arrangement of the magnetic field values during this study. The magnetic fields between the individual trials did not interfere with each other. The letters represent the following: a—magnet dummy, b—location of the eggs/larvae exposed to the magnetic field, c—crystallizer, d—tested magnetic field value affecting the eggs/larvae, and e—magnets generating the magnetic field.

The egg incubation and larval rearing were divided into two stages. From five spawning pairs of each species, 1200 eggs were collected at a time and divided into four batches. Each batch consisted of 300 eggs. The experiment was conducted on a total of 6000 larvae for each species. The eggs were incubated in separate 500 mL crystallizers filled with water from the broodstock tank, ensuring that the incubated eggs were maintained in physicochemical conditions identical to those of their native environment. Each crystallizer was aerated to provide constant water circulation. The temperature in the crystallizers was maintained at a constant level of 26 ± 0.5 °C. Each crystallizer containing eggs was exposed to a magnetic field of a different flux density: trial 1—1 mT (a value 20 times greater than the Earth’s magnetic field), trial 2—3 mT (a value 60 times greater than the Earth’s magnetic field), and trial 3—5 mT (a value 100 times greater than the Earth’s magnetic field). After placing of the crystallizers containing the eggs, the magnetic field strength was measured using a magnetic field meter. The control group (C) consisted of eggs placed outside the range of the generated magnetic field—exposed only to the Earth’s natural magnetic field (Figure 1). The eggs were incubated under these conditions until hatching (no manipulations were performed during this period).

Observations and recordings of the larvae were carried out using Nikon 2000SE (Tokyo, Japan) and Carl Zeiss Stereo Discovery V12 (Jena, Germany) microscopes equipped with image acquisition and analysis software. The observations began when the larvae emerged from the egg envelopes. Changes during the larval period were analyzed and recorded once daily at the same time over the first four days of life until 3/4 of the yolk sac volume had been resorbed. Day 0 was defined as the day of larval hatching, when 100% of the larvae had emerged from the egg envelopes. The observations were concluded after 120 h of larval life, at the point when the larvae began to reach approximately 3/4 yolk sac resorption. For each observation, 11 larvae were randomly selected from each crystallizer and then transferred to a separate crystallizer, as they were no longer included in further studies (handling the larvae induced stress that could have interfered with the experimental outcomes). During the experiment, the number of dead and deformed larvae was recorded and then removed from the experimental setup each time. To calculate the survival rate (ls), the following formula was used: ls = 100% − (a × 100/b), where a is the number of dead larvae and b is the total number of larvae in the experiment. To calculate the percentage of deformities (ld), the following formula was used: ld = a × 100/b, where a is the number of deformed larvae and b is the total number of larvae in the experiment. The criteria used to assess the deformed larvae included deformities of the notochord, head, and yolk sac.

**Figure 2:**
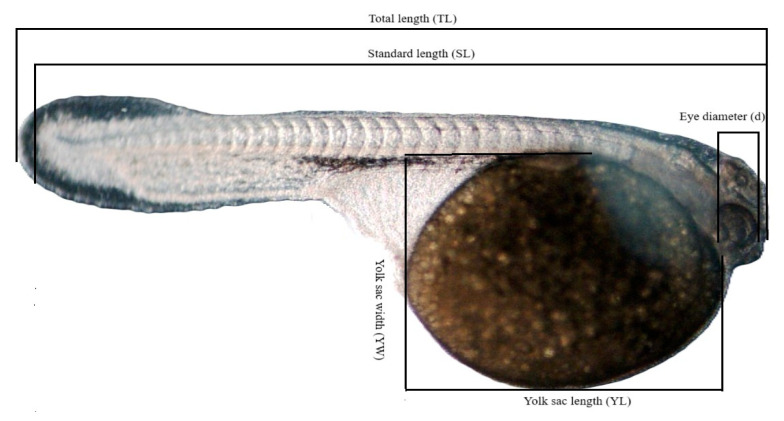
Jaguar cichlid larva with labels: total length (TL), standard length (SL), yolk sac length (YL), yolk sac width (YW), and eye diameter (d). Larva at 72 h post-hatching, larval length—5.7 mm.

After the experiment, the collected images were analyzed using MultiScan program, and larval measurements were taken: total length (TL), standard length (SL), yolk sac length (YL), yolk sac width (YW), and eye diameter (d) (Figure 2). Due to the shapes of the yolk sacs in the larvae of the studied species, the formula for the volume of a prolate ellipsoid was used: V = π/6 × YL × 〖YW〗^2^. The length measurements were averaged (mean ± SD) and then compared. The mean values were compared between the first and the last day, as well as between different samples within each day. The Bonferroni test was used for multiple comparisons, as it provides a high level of confidence in avoiding type I errors. Statistical analyses were performed using Statistica software, version 13.3.

## 3. Results

### 3.1. Larval Size

**Figure 3:**
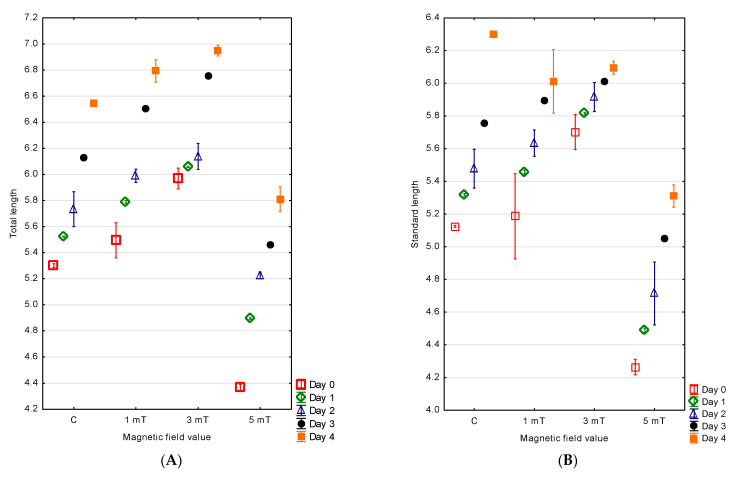
Changes in total length (**A**) and standard length (**B**) in *P. managuensis*.

The hatching of the jaguar cichlid larvae occurred over an extended period. Fifty percent of the larvae hatched within 3 h from the moment the first larva emerged from the egg envelope, while the last individuals hatched after 7 h across all experimental groups. The largest larvae were observed in the group exposed to the magnetic field of 3 mT, with a mean total length of 5.97 ± 0.04 mm and a standard length of 5.70 ± 0.05 mm. Smaller larvae were noted in the 1 mT group, with a total length of 5.50 ± 0.07 mm and a standard length of 5.19 ± 0.13 mm. In the control group—not exposed to a magnetic field—the larvae measured, on average, 5.30 ± 0.01 mm in total length and 5.13 ± 0.01 mm in standard length. The smallest larvae were observed in the group exposed to the strongest magnetic field (5 mT), where the total length was 4.37 ± 0.01 mm and the standard length was 4.26 ± 0.02 mm. The differences between all experimental groups were statistically significant (*p* < 0.001) (Figure 3A,B).

The measurements taken on the fourth (final) day of observation confirmed continued differences in the larval size. The largest individuals were again observed in the 3 mT group, with a total length of 6.95 ± 0.02 mm and a standard length of 6.10 ± 0.02 mm. In the 1 mT group, the total length reached 6.79 ± 0.04 mm and the standard length 6.01 ± 0.10 mm. Slightly smaller larvae were recorded in the control group, with a total length of 6.55 ± 0.01 mm and a standard length of 6.30 ± 0.01 mm. The smallest larvae remained those from the group exposed to the highest magnetic field (5 mT), with a total length of 5.81 ± 0.05 mm and a standard length of 5.31 ± 0.03 mm. The differences between all experimental groups were statistically significant (*p* < 0.001) (Figure 3A,B).

Differences were also found in the larval growth over the first four days of life among the experimental groups. The greatest increase in body length was observed in the 5 mT group—1.44 mm. Slightly lower growth was recorded in the 1 mT group (1.30 mm) and in the control group (1.24 mm). The smallest growth was noted in group 2 (3 mT), amounting to 0.98 mm. The differences between all experimental groups were statistically significant (*p* < 0.001).

**Figure 4:**
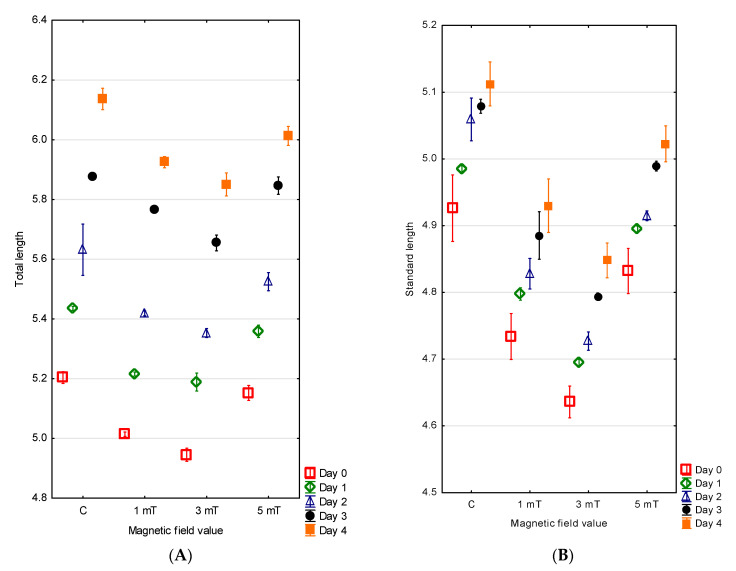
Changes in total length (**A**) and standard length (**B**) in *A. rivulatus*.

The hatching in the green terror was also extended over time. Fifty percent of the larvae hatched within 3 h of the emergence of the first individual from the egg envelope, while the last larvae hatched after 7 h across all experimental groups. The largest larvae were observed in the control group, with a mean total length of 5.20 ± 0.01 mm and a standard length of 4.93 ± 0.03 mm. Slightly smaller larvae were recorded in group 3 (5 mT), with a total length of 5.15 ± 0.01 mm and a standard length of 4.83 ± 0.02 mm. In group 1 (1 mT), the green terror larvae reached a total length of 5.01 ± 0.01 mm and a standard length of 4.73 ± 0.02 mm. The smallest larvae were observed in group 2 (3 mT), with a total length of 4.95 ± 0.01 mm and a standard length of 4.64 ± 0.01 mm (Figure 4A,B). The differences between all experimental groups were statistically significant (*p* < 0.001).

The measurements taken on the fourth (final) day of observation showed that the differences in larval size persisted. The largest larvae were found in the control group, with a total length of 6.14 ± 0.02 mm and a standard length of 5.11 ± 0.02 mm. Slightly smaller individuals were recorded in group 3 (5 mT), with a total length of 6.01 ± 0.02 mm and a standard length of 5.02 ± 0.01 mm. Even smaller larvae were noted in group 1 (1 mT), with a total length of 5.93 ± 0.01 mm and a standard length of 4.93 ± 0.02 mm. The smallest larvae were again observed in group 2 (3 mT), where the total length was 5.85 ± 0.02 mm and the standard length 4.85 ± 0.01 mm. The differences between all experimental groups were statistically significant (*p* < 0.001) (Figure 4A,B).

Differences were also noted in the larval growth during the first four days of life across the experimental groups. The highest growth values were recorded in group 1 (1 mT) and group 2 (3 mT), with increases of 0.91 mm and 0.97 mm, respectively. Lower growth was observed in the control group (0.87 mm), while the lowest growth occurred in group 3 (5 mT), amounting to 0.86 mm. The differences between all experimental groups were statistically significant (*p* < 0.001).

### 3.2. Yolk Sac

**Figure 5:**
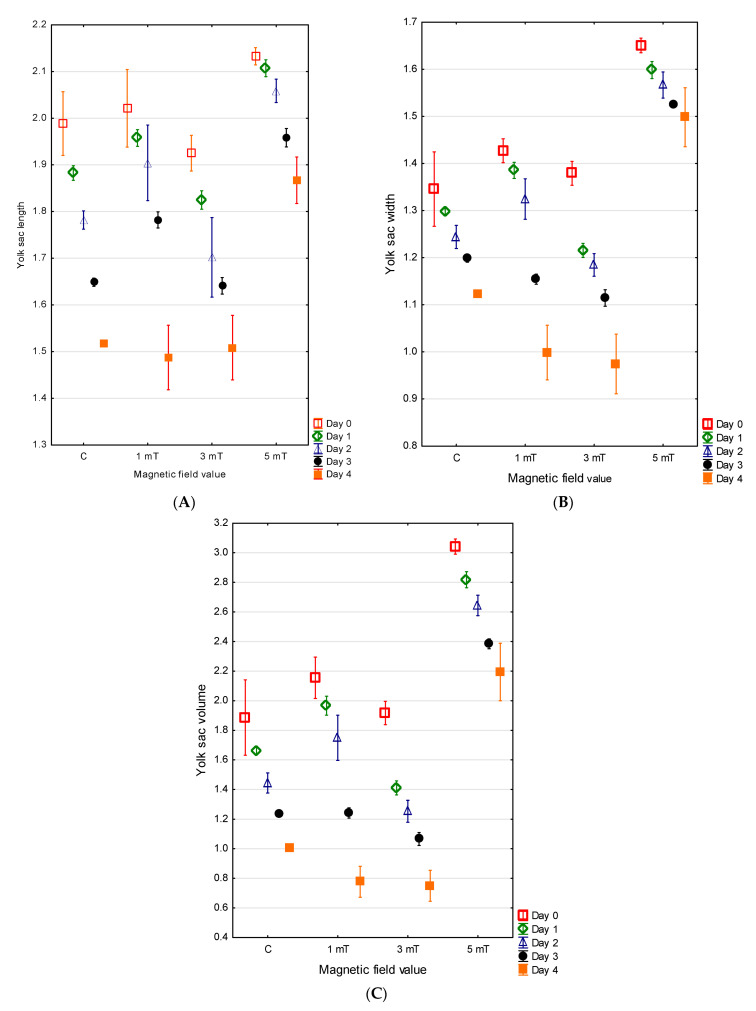
Changes in yolk sac length (**A**), width (**B**), and volume (**C**) in *P. managuensis*.

At the time of hatching, the jaguar cichlid larvae possessed large, oval yolk sacs. The largest yolk sac, measuring, on average, 2.13 ± 0.01 mm in length and 1.65 ± 0.01 mm in width (V = 3.04 ± 0.03 mm^3^), was observed in group 3 (5 mT). In group 1 (1 mT), the yolk sacs measured an average of 2.02 ± 0.04 mm in length and 1.43 ± 0.01 mm in width (V = 2.15 ± 0.07 mm^3^). Slightly smaller yolk sacs in the newly hatched jaguar cichlid larvae were recorded in group 2 (YL = 1.93 ± 0.02 mm, YW = 1.38 ± 0.41 mm, V = 1.92 ± 0.04 mm^3^). The smallest yolk sacs were found in the control group (YL = 1.99 ± 0.03 mm, YW = 1.35 ± 0.04 mm, V = 1.88 ± 0.13 mm^3^). The differences between all experimental groups were statistically significant (*p* < 0.001) (Figure 5A–C).

No direct relationship was observed between the rate of larval body growth and the rate of yolk sac resorption. The largest yolk sac on the fourth day of development in the jaguar cichlid larvae was again recorded in group 3 (5 mT) (YL = 1.87 ± 0.03 mm, YW = 1.50 ± 0.03 mm, V = 2.19 ± 0.10 mm^3^), and the yolk sac resorption in this group progressed the slowest. On the fourth day of larval life, the larvae from the control group retained yolk sacs with the dimensions YL = 1.52 ± 0.01 mm, YW = 1.12 ± 0.01 mm, and V = 1.02 ± 0.01 mm^3^ and had resorbed 46% of the yolk sac. The most rapid resorption occurred in the larvae from group 1 (1 mT) and group 2 (3 mT). In group 1 (1 mT), the larvae resorbed 64% of the yolk sac (YL = 1.49 ± 0.04 mm, YW = 0.99 ± 0.03 mm, V = 0.78 ± 0.05 mm^3^), while in group 2, 61% was resorbed (YL = 1.51 ± 0.04 mm, YW = 0.97 ± 0.03 mm, V = 0.75 ± 0.05 mm^3^). The differences between all experimental groups were statistically significant (*p* < 0.001) (Figure 5A–C).

**Figure 6:**
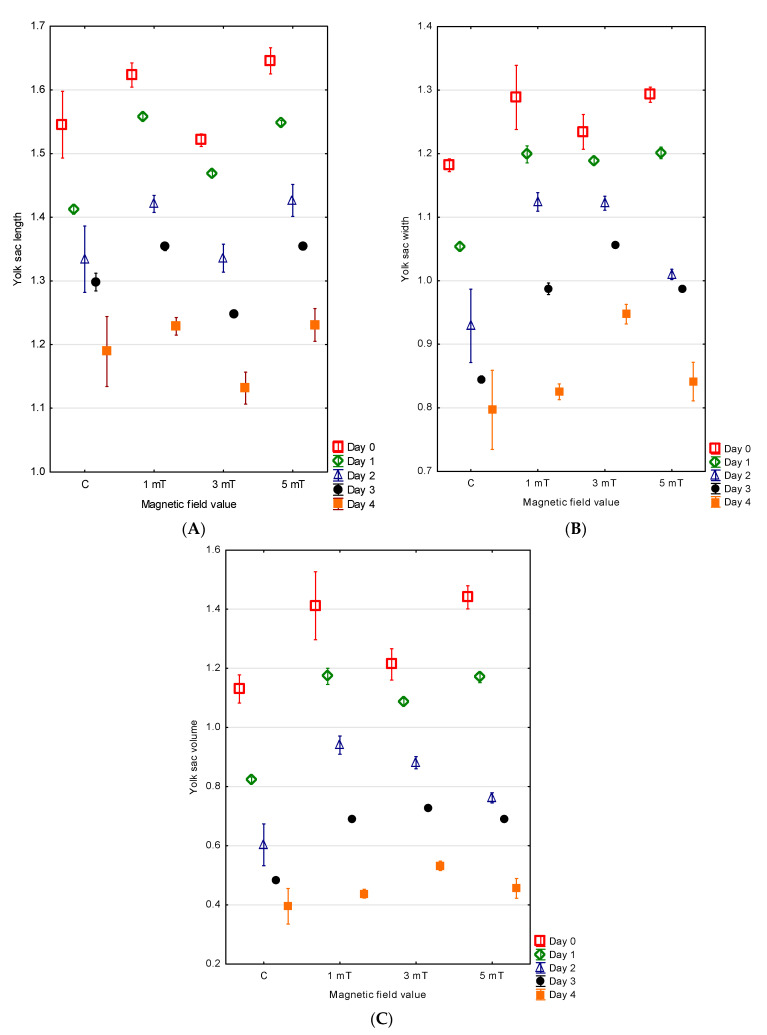
Changes in yolk sac length (**A**), width (**B**), and volume (**C**) in *A. rivulatus*.

At hatching, the green terror larvae possessed large, oval yolk sacs. The largest yolk sac by volume was observed in group 3 (5 mT), with mean measurements of YL = 1.65 ± 0.01 mm, YW = 1.29 ± 0.01 mm, and V = 1.44 ± 0.02 mm^3^. A slightly smaller yolk sac was found in group 1 (1 mT), with YL = 1.62 ± 0.01 mm, YW = 1.29 ± 0.03 mm, and V = 1.41 ± 0.06 mm^3^. In group 2 (3 mT), the yolk sacs were somewhat smaller, with a mean length of 1.52 ± 0.01 mm, width of 1.23 ± 0.01 mm, and volume of 1.21 ± 0.03 mm^3^. The smallest yolk sac was recorded in the control group, with YL = 1.55 ± 0.03 mm, YW = 1.18 ± 0.01 mm, and V = 1.13 ± 0.02 mm^3^. The differences between all experimental groups were statistically significant (*p* < 0.001) (Figure 6A–C).

No direct relationship was observed between the rate of body growth and the rate of yolk sac resorption. The largest yolk sac on the fourth day of development was found in group 2 (3 mT) (YL = 1.13 ± 0.01 mm, YW = 0.95 ± 0.01 mm, V = 0.53 ± 0.01 mm^3^). The larvae in this group resorbed the yolk sac most slowly—on day four, it still constituted 56% of the initial volume. Slightly smaller yolk sacs were noted in group 3 (5 mT) (YL = 1.23 ± 0.01 mm, YW = 0.84 ± 0.02 mm, V = 0.46 ± 0.02 mm^3^), with the larvae resorbing 68% of the yolk sac content. In group 1 (1 mT), the yolk sac dimensions were YL = 1.23 ± 0.01 mm, YW = 0.83 ± 0.01 mm, and V = 0.44 ± 0.01 mm^3^, with 69% of the yolk content resorbed. The smallest yolk sac on day four was observed in the control group (YL = 1.19 ± 0.03 mm, YW = 0.80 ± 0.03 mm, V = 0.40 ± 0.03 mm^3^), where 65% of the initial yolk sac volume had been resorbed. The differences between all experimental groups were statistically significant (*p* < 0.001) (Figure 6A–C).

### 3.3. Eyes

The measurements of the eye diameter in the developing jaguar cichlid larvae indicated that the individuals developing in magnetic fields of different flux densities exhibited variable eye sizes. The largest eye diameter in the newly hatched larvae was observed in group 2 (3 mT)—0.48 ± 0.02 mm. Slightly smaller eyes were found in group 1 (1 mT)—0.43 ± 0.04 mm. In the control group, the mean eye diameter was 0.37 ± 0.02 mm. The smallest eye diameter was recorded in group 3 (5 mT)—0.25 ± 0.01 mm.

**Figure 7:**
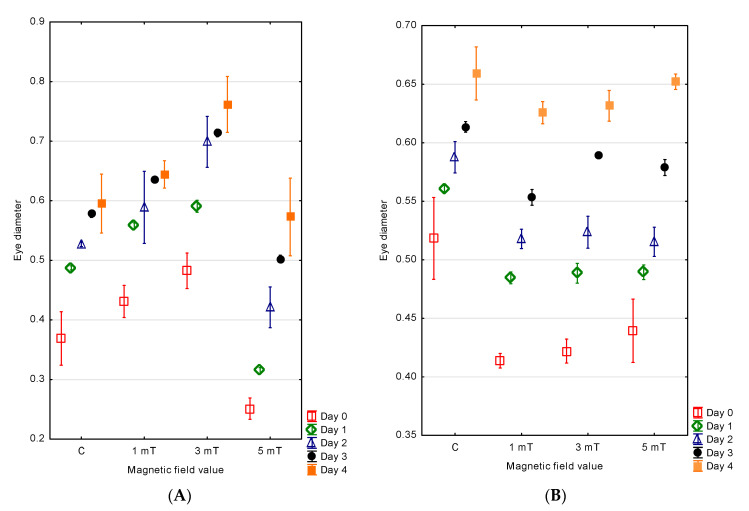
Changes in eye diameter in *P. managuensis* (**A**) and *A. rivulatus* (**B**).

The eye diameter growth was correlated with the larval body growth. On the fourth day of development, the larvae from group 2 (3 mT) had the largest eyes (d = 0.76 ± 0.02 mm). In group 1 (1 mT), the eye diameters were approximately 15% smaller and measured 0.64 ± 0.01 mm. In the control group, the diameters were 22% smaller compared with group 2 and reached 0.59 ± 0.03 mm. The smallest eyes were recorded in group 3 (5 mT)—0.57 ± 0.05 mm (Figure 7A).

In the green terror larvae, the magnetic field exposure also influenced the eye diameter. The largest eyes at the time of hatching were observed in the control group (d = 0.52 ± 0.02 mm). Smaller eyes were recorded in group 3 (5 mT) (d = 0.44 ± 0.01 mm), and even smaller in group 2 (3 mT) (d = 0.42 ± 0.01 mm). The smallest eyes were found in group 1 (1 mT), with diameters of 0.41 ± 0.01 mm.

The eye diameter increased in parallel with the larval body growth. On day four of development, the larvae from the control group had the largest eyes (d = 0.66 ± 0.01 mm). In group 3 (5 mT), the eye diameters were 7% smaller (d = 0.65 ± 0.01 mm). The eyes in group 2 (3 mT) were 10% smaller (d = 0.63 ± 0.01 mm), while the smallest eyes were observed in group 1 (1 mT): 11% smaller (d = 0.63 ± 0.01 mm) (Figure 7B).

### 3.4. Survival and Larval Deformities

The magnetic field influenced both the larval survival and the incidence of deformities in the two studied species. After four days of larval development, the highest survival rate in the jaguar cichlids was observed in group 1 (1 mT), reaching 92%. A slightly lower rate of 90% was recorded in group 2 (3 mT), and the lowest—87%—in group 3 (5 mT). In the control group, which was not exposed to the magnetic field, the survival was the lowest, at 85%.

**Figure 8:**
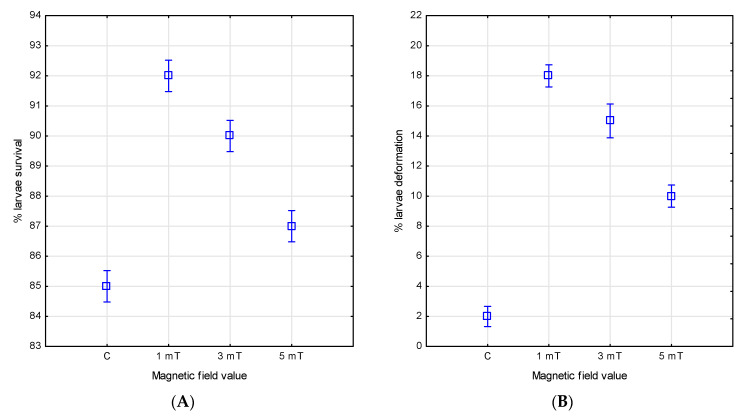
Survival (**A**) and number of deformities (**B**) in *P. managuensis* larvae.

**Figure 9:**
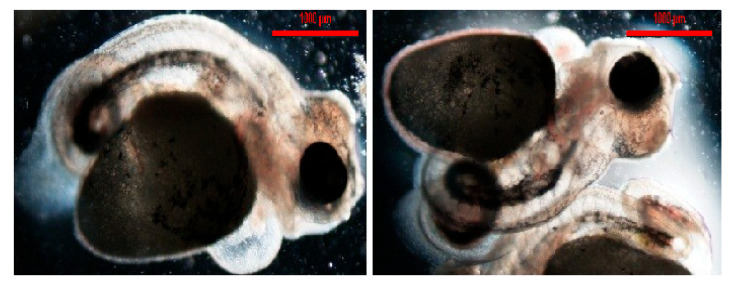
An example of deformed larvae in *P. managuensis*. Deformed larva 48 h post-hatching.

Analysis of the deformity rates showed that only 2% of the individuals were deformed in the control group. In contrast, higher deformity rates were observed in all magnetic-field-exposure groups. The highest rate of deformities was found in group 1 (1 mT)—18%, followed by group 2 (3 mT)—15%, and the lowest in group 3 (5 mT)—10% (Figure 8A,B and Figure 9).

The magnetic field also affected the survival and incidence of the deformities in the green terror larvae. After four days of observation, the highest survival rate—90%—was recorded in both the control group and group 2 (3 mT), with no statistically significant differences between them. In group 3 (5 mT), the larval survival reached 88%; this result differed significantly from the control group and group 2 (*p* < 0.005), as well as from group 1 (*p* < 0.001). The lowest survival rate was observed in group 1 (1 mT)—85%, with statistically significant differences compared with all other groups (control, group 2, and group 3—*p* < 0.001).

**Figure 10:**
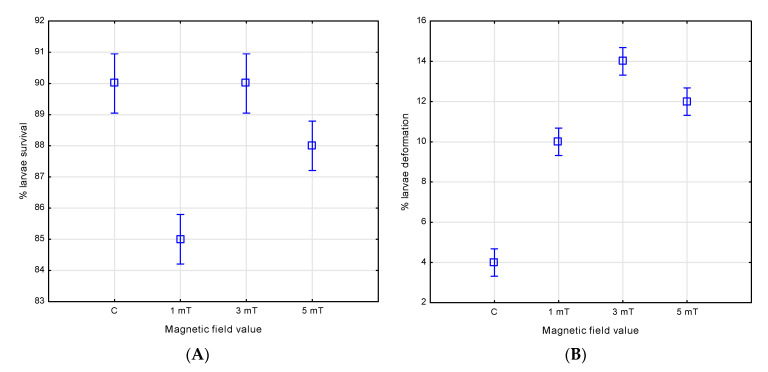
Survival (**A**) and number of deformities (**B**) in *A. rivulatus* larvae.

Analysis of the larval deformities showed that only 4% of the larvae were deformed in the control group. In contrast, higher deformity rates were observed in the groups exposed to the increased magnetic flux densities: 14% in group 2 (3 mT), 12% in group 3 (5 mT), and 10% in group 1 (1 mT). The differences between all experimental groups were statistically significant (*p* < 0.001) (Figure 10A,B).

## 4. Discussion

The present study expands the current knowledge on the effects of magnetic fields of selected flux densities (1 mT, 3 mT, and 5 mT) on the early ontogeny of two cichlid species—the jaguar cichlid (*P. managuensis*) and the green terror (*A. rivulatus*). The results obtained provide a better understanding of both the beneficial and adverse effects of magnetic fields of varying flux density on the development of these species—from hatching to the stage when three-quarters of the yolk sac had been resorbed. This study focused on evaluating how magnetic fields of different flux densities affect changes in body and eye size, the rate of yolk sac resorption, survival, and the frequency of larval deformities.

Although both taxa belong to the same family, the experimental results demonstrated distinct responses to the magnetic stimulus, likely due to differences in the environmental requirements, body size, biology, and native habitats. The magnetic field affected the growth rate (and thus the size) of *P. managuensis* but did not produce a similar effect in *A. rivulatus*. Continuous exposure to the magnetic field throughout the embryonic development influenced the sizes of the hatched larvae and their yolk sac dimensions in *P. managuensis*. The most favorable effect was observed in the 3 mT group, where the larvae were the largest and had the smallest yolk sac volumes. The least favorable outcomes were noted in the 5 mT group, where larvae were approximately 25% smaller than those in the control group and possessed the largest yolk sacs.

Depending on the magnetic flux density, significant differences were found in both the body growth rate and the yolk sac resorption rate in *P. managuensis*. Compared with the control group (without magnetic exposure), the larvae exposed to different flux densities displayed varied growth dynamics, which was reflected in differences in both body size increments and yolk sac volume reduction. In the 5 mT group, the *P. managuensis* larvae exhibited the fastest growth, whereas in the 3 mT group, despite having the largest overall size, the growth rate was the slowest. The influence of magnetic field exposure on growth and yolk sac resorption was also reflected in the eye size: in the 3 mT group, the eye diameter increased the fastest in the *P. managuensis* larvae.

An interesting phenomenon was observed in the *A. rivulatus* larvae subjected to the magnetic field exposure. In all experimental groups, these larvae were smaller in body size, had larger yolk sacs, and smaller eyes compared with the control group. Despite their smaller size, the larvae in all magnetic field groups showed a faster growth rate than the control group. The lack of a pronounced response to the magnetic field exposure in this species may be related to its biology—in its natural environment, *A. rivulatus* inhabits only certain streams in South America [16,17,18,19,20] and likely does not engage in long-distance migrations to spawning grounds guided by a magnetic field, as is the case with migratory fish such as salmonids [42,43,44,45,46]. As such, this species may not have evolved magnetoreceptive capabilities. Further research on the fry and adults of this species would be valuable in confirming this hypothesis.

Contrary to the findings of Fey [33], who reported that magnetic field exposure at 10 mT had no effect on the growth or body mass of rainbow trout (*O. mykiss*) larvae but did affect the rate of yolk sac resorption, the present study showed that magnetic field exposure at 5 mT had a negative impact on the *P. managuensis* larval size. Magnetic field effects on larval size have also been documented in roaches (*R. rutilus*), where exposure has led to changes in both body size and mass [32]. Additionally, Formicki and Winnicki [24] found that larvae of sea trout (*Salmo trutta*) and rainbow trout (*O. mykiss*) incubated under magnetic field exposure achieved greater sizes and body masses than those developed without magnetic exposure. Similar results were observed in the *P. managuensis* larvae in the present study: individuals from the 1 mT and 3 mT groups were larger at hatching than those in the control group. Conversely, the *A. rivulatus* larvae exposed to magnetic fields were smaller than the control larvae.

This experiment also provided important insights into the influence of magnetic fields on larval survival and deformities. The magnetic field exposure affected both species studied—though in markedly different ways. In *P. managuensis*, the survival increased with decreasing magnetic field strength. In contrast, for *A. rivulatus*, the survival was lower in all groups except the 3 mT group when compared with the control. These patterns were mirrored in the deformity rates: in both species, higher survival was associated with a higher proportion of deformities. This suggests that magnetic field exposure may allow the survival of individuals with developmental defects that would not persist under natural conditions.

## 5. Conclusions

A comprehensive analysis of the results indicates that the effects of magnetic field exposure depend both on the magnetic flux density applied and on the species studied. Increased survival of the *P. managuensis* larvae was observed in the groups exposed to the magnetic field; however, this was accompanied by a higher incidence of deformities compared with the control group. In contrast, for the *A. rivulatus*, the highest survival rates were recorded in the control group and in the group exposed to the 3 mT magnetic field, although even in these cases, an increased number of deformities was observed in the magnetic-field-exposure groups.

The findings from this study may hold significant implications for ornamental fish aquaculture, where appropriately calibrated magnetic field exposure could potentially enhance larval growth and survival, thereby reducing production losses. Nevertheless, in order to fully understand the mechanisms underlying the effects of magnetic fields on fish larval development, further research is needed. Future studies should include longer observation periods and assess the influence of magnetic fields on the subsequent developmental stages of fish.

## Data Availability

The data generated and analyzed during the current study are available from the corresponding author upon reasonable request. The data are not publicly available due to the excessive amount of collected data.
